# Role of Neurofilament Light Chain as a Potential Biomarker for Alzheimer's Disease: A Correlative Meta-Analysis

**DOI:** 10.3389/fnagi.2019.00254

**Published:** 2019-09-13

**Authors:** Mei Jin, Li Cao, Yan-ping Dai

**Affiliations:** Department of Neurology, Nangang Branch, Heilongjiang Provincial Hospital, Harbin, China

**Keywords:** Alzheimer's disease, neurofilament light, diagnosis, biomarker, correlation

## Abstract

Neurofilament light (NFL) is a putative biomarker of neurodegeneration. This study evaluates the correlative association of NFL with Alzheimer's disease (AD) indices. Relevant studies were identified after a literature search in electronic databases and study selection was based on pre-determined eligibility criteria. Correlation coefficients between NFL levels and important AD indices reported by individual studies were pooled as z-scores. Meta-regression analyses were performed to evaluate the relationships between important covariates. Data from 38 studies (age 68.3 years [95% confidence interval (CI): 65.7, 70.9]; 54 % [95% CI: 50, 57] females) were used. Meta-analyses of correlation coefficients reported by the included studies showed that NFL levels in blood and cerebrospinal fluid (CSF) correlated well (*r* = 0.59 [95% CI: 0.45, 0.71]; *p* < 0.0001). NFL levels correlated with MMSE score (*r* = −0.345 [95% CI: −0.43, −0.25]; *p* = 0.0001), and age (*r* = 0.485 [95% CI: 0.35, 0.61]; *p* = 0.00001). CSF NFL levels correlated with total tau (t-tau; *r* = 0.39 [95% CI: 0.27, 0.50]; *p* = 0.0001), phosphorylated tau (p-tau; *r* = 0.34 [95% CI: 0.19, 0.47]; *p* = 0.00001), and neurogranin (*r* = 0.25 [95% CI: 0.12, 0.37]; *p* = 0.001) but not with beta amyloid (Aβ) (*r* = 0.00 [95%CI: −0.13, 0.12]; *p* = 0.937). In meta-regression, MMSE scores were associated inversely with blood NFL (metaregression coefficient (MC) −0.236 [95% CI:−0.40, −0.072; *p* = 0.008), and age (MC) −0.235 [−0.36, −0.11]; *p* = 0.001) and positively with CSF Aβ-42 (MC 0.017 [0.010, 0.023]; *p* = 0.00001). NFL has good correlations with t-tau, and p-tau in CSF and CSF NFL levels correlates well with blood NFL levels. These results show that NFL can be a useful biomarker for improving diagnosis and predicting prognosis in AD patients especially if age weighted.

## Introduction

Alzheimer's disease (AD) is a neurodegenerative disease with cognitive deficits and progressive neural atrophy that develop with the accumulation of beta-amyloid (Aβ) and tau proteins in the brain (Ballard et al., 2011). AD is frequently associated with dementia and frailty in late age. Milder symptoms such as memory problems are usually followed by cognitive impairment that adversely affect complex daily life activities (Kukull and Bowen, 2002). AD is usually diagnosed at a time when many brain areas already have neuronal loss and neuropathological lesions (Lane et al., 2018).

Whereas, between 2000 and 2013, stroke-, heart disease-, and prostate cancer-related mortality decreased by 23, 14, and 11%, respectively, AD-caused deaths increased by 71% (Alzheimer's Association, 2016). In the USA alone, the prevalence of AD is 5.4 million where in 2015 over 15 million family or other caregivers devoted approximately 18.1 billion hours of care to patients with dementia, including AD with dementia (ADD) which is the most common form of dementia (Alzheimer's Association, 2016). Worldwide, over 50 million people (mostly over 65 years of age) have some form of dementia of which AD dementia accounts for 60–70%. AD is the sixth leading cause of death in the US, and by 2050 approximately 135 million people are estimated to be affected by AD (Anonymous, 2016).

AD is mainly characterized by Aβ deposition forming plaques when amyloid fibrils are accumulated in the extracellular space. In addition to Aβ, tau and phosphorylated tau (p-tau) protein are biomarkers of neuronal injury. The presence of both these biomarker increases the probability of AD diagnosis (Mantzavinos and Alexiou, 2017). A higher degree of amyloid plaques causes lowered Aβ levels in CSF. On the other hand, a higher degree of neurofibrillary tangling leads to higher tau CSF levels. Aβ, tau, phosphorylated-tau (p-tau) or their ratios have associations with neurofibrillary tangles and neuritic plaques. Additionally, the markers of inflammatory processes, oxidative stress and blood-brain barrier damage may also reflect the state of disease pathogenicity (Rosenmann, 2012). To date, CSF levels of total tau (t-tau), p-tau, and Aβ-42 (a 42 amino acid chain of Aβ) are established biomarkers of AD (Olsson et al., 2016). The combined use of Aβ42, p-tau and t-tau for the diagnosis of AD yields 85–95% sensitivity and specificity (Hoglund et al., 2015).

Neurofilament is an axonal cytoskeletal protein found only in neurons. Of the 4 subunits (light, medium, heavy, and alpha-internexin), neurofilament light (NFL) is the most abundant. In addition to AD, elevated CSF NFL levels are found in other neurodegenerative diseases including vascular dementia and frontotemporal dementia (FTD). NFL is a putative biomarker of neurodegeneration, especially for subcortical neurodegeneration. NFL is also suggested to be capable of measuring neurodegeneration in AD patients (Petzold et al., 2007; Zetterberg and Blennow, 2018).

However, whether NFL measurement in AD patients can improve diagnostic efficiency is not clear. One way to appraise this phenomenon could be to examine the strength of the relationship between NFL and other biomarkers and cognitive markers of AD. The aim of the present study was to evaluate the relationships between NFL and (1) other AD biomarkers and (2) the cognitive performance of AD patients by (a) performing meta-analyses of correlation coefficients reported by individual studies, and (b) examining correlative relationships between NFL and other biomarkers/indices from baseline data reported by the individuals studies fulfilling the eligibility criteria of the present meta-analysis.

## Materials and Methods

### Eligibility Criteria

The inclusion criteria were that the study (a) estimated NFL and other related AD biomarkers in the cerebrospinal fluid (CSF) or blood of AD patients, (b) reported correlation coefficients quantifying the relationship between NFL levels and AD biomarkers, and (c) reported the MMSE scores of AD patients or their relationships with NFL concentrations. Studies were excluded if they reported outcomes of other resembling disorders such as FTD, vascular dementia, or dementia with Lewy bodies (DLB) or if they focused on neurofilament high chain. Additionally, articles reporting the relationship of NFL with other biomarkers or related factors as regression coefficients but not as correlation coefficients were excluded.

### Literature Search

Electronic databases (Embase, Google Scholar, Ovid SP, and PubMed) were searched for research articles using relevant keywords in logical combinations. For this purpose, the phrase “Alzheimer's disease-neurofilament light-biomarker” was used with several terms including serum, plasma, cerebrospinal fluid, CSF, mini-mental state examination, MMSE, cognitive impairment, dementia, memory, correlation, diagnosis, risk, and relationship. Cross references of important research papers (relevant review articles, eligible studies, and software-suggested articles) were also screened. The literature search, which was restricted to English language as publication medium, was completed by May 2019.

### Data and Analyses

Data were obtained from the published research articles of the selected studies. Demographic, molecular, serological, and mental function evaluation data; correlation coefficients; blood and CSF levels of biomarkers; and other related information were extracted from articles and were organized in datasheets. Correlation coefficients between NFL and other biomarkers or factors were first converted into Fisher's z scores to be used in the meta-analyses which were performed under a random effects model with Stata software (Stata Corporation, Texas, USA). For this purpose, z scores along with respective standard errors were pooled to obtain overall or subgroup effect sizes as the inverse variance weighted average of an individual study z score. These z-scores were then back-transformed to correlation coefficients. Statistical heterogeneity (between-study inconsistency in outcomes) was evaluated by using the I^2^ index.

## Results

**Figure 1:**
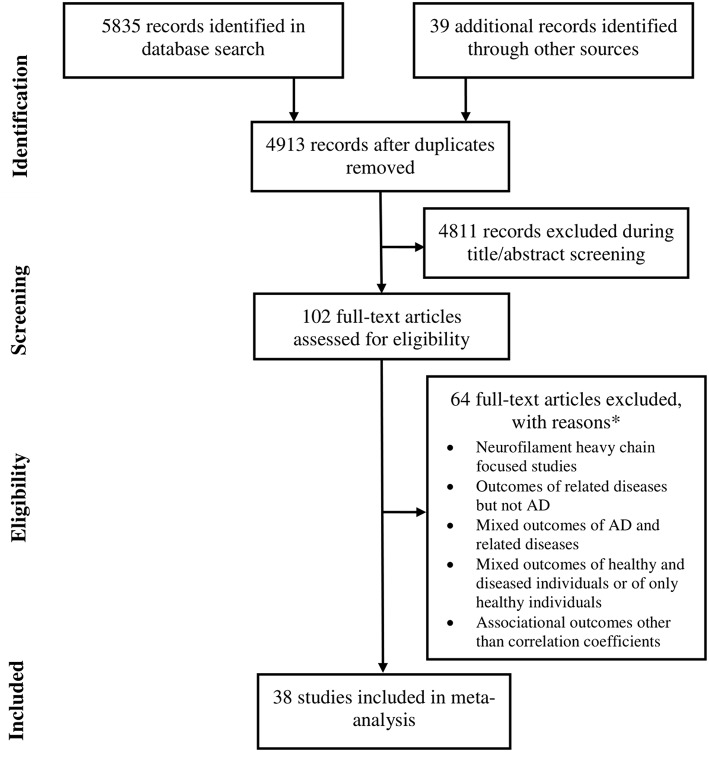
A flowchart of study screening and selection process.

Data were extracted from 38 studies (Sjögren et al., 2001; de Jong et al., 2007; Pijnenburg et al., 2007; Bjerke et al., 2011; Kester et al., 2012; Gaiottino et al., 2013; Landqvist Waldö et al., 2013; Skillbäck et al., 2013; Evered et al., 2016; Melah et al., 2016; Fialova et al., 2017; Howell et al., 2017; Jensen et al., 2017; Lista et al., 2017; Pereira et al., 2017; Weston et al., 2017, 2019; Zhou et al., 2017; Bettcher et al., 2018; Chatterjee et al., 2018; Fortea et al., 2018; Hampel et al., 2018; Lewczuk et al., 2018; Lin et al., 2018; Merluzzi et al., 2018; Olsson et al., 2018; Paterson et al., 2018; Sánchez-Valle et al., 2018; Steinacker et al., 2018; Vogt et al., 2018; Zerr et al., 2018; Ashton et al., 2019; Bos et al., 2019; Lleó et al., 2019; Marchegiani et al., 2019; Mattsson et al., 2019; Niikado et al., 2019) which were selected by following eligibility criteria (Figure 1). Except for 2 longitudinal studies all others were cross sectional in design. Overall, data were available for 8678 individuals with AD. The average age of these patients was 68.3 years [95% confidence interval (CI): 65.7, 70.9] and 53.5 % [95% CI: 49.9, 57.07] of these individuals were female (Table 1).

**Table 1:** Characteristics of the included studies.

**References**	***n***	**AD type**	**Age**	**% females**	**CSF NFL**	**Blood NFL**	**Aβ-42**	**T-tau**	**P-tau**
Ashton et al., 2019	57	AD	83 ± 3.2	61.4		42 ± 7			
Bettcher et al., 2018	173	AD	63.9 ± 7.1	65	796 ± 636		738 ± 202	340 ± 169	47.4 ± 18
Bjerke et al., 2011	30	AD	68 ± 3.2	63.33	1, 400 ± 563		363 ± 130	580 ± 374	82 ± 40
Bos et al., 2019	180	ADD	70.5 ± 8.7	52	1, 756 ± 2, 893		237 ± 113	673 ± 465	81 ± 37
Chatterjee et al., 2018	100	AD	78 ± 5.6	68		36 ± 19			
de Jong et al., 2007	37	EAD	61 ± 4.9	59.46			365 ± 152	565 ± 531.4	647 ± 61
de Jong et al., 2007	33	LAD	76 ± 6.1	60.61			419 ± 198	86 ± 667.7	89 ± 66
Evered et al., 2016	59	AD	70.4 ± 7	68	772 ± 355		759 ± 310	327 ± 164	44.5 ± 356
Fialova et al., 2017	20	AD	71 ± 7	85	3, 000 ± 1, 436	7 ± 35		700 ± 447	62 ± 30
Fortea et al., 2018	22	ADD-DS	54 ± 11.6	43	1, 100 ± 325	23 ± 12	392 ± 143	853 ± 377	95 ± 46
Gaiottino et al., 2013	20	AD	72.5 ± 7.3	65	1, 396 ± 580	31 ± 14			
Hampel et al., 2018	35	ADD	73 ± 8.1	68.57	1, 483 ± 623		424 ± 159	496 ± 268.6	83 ± 44
Howell et al., 2017	27	AD	71.4 ± 9.1	59.26	1, 202 ± 102		192 ± 140	78 ± 34	30 ± 16
Jensen et al., 2017	26	AD	68.9 ± 8.1	26.92	1, 495 ± 635				
Kester et al., 2012	68	AD	65 ± 7	45.59	5, 600 ± 4, 400		263 ± 83	156 ± 87	43 ± 26
Lewczuk et al., 2018	25	ADD	70.8 ± 7.6	61		49 ± 28	536 ± 114	558 ± 178	89.9 ± 18
Lewczuk et al., 2018	33	AD-MCI	70.8 ± 7.6	61		38 ± 16	585 ± 116	631 ± 214	101 ± 30
Lin et al., 2018	119	AD	77.3 ± 5.1	53		33 ± 26			
Lista et al., 2017	35	AD	73 ± 2.3	50	1, 483 ± 623		424 ± 159	496 ± 269	83 ± 44
Lleó et al., 2019	110	AD	68.5 ± 8.5	42.73	1, 647 ± 1, 573		316 ± 192	759 ± 432	81 ± 37
Marchegiani et al., 2019	70	AD	77 ± 7.7	62.86	1, 333 ± 2, 047		385 ± 322	447 ± 957.8	64 ± 70
Mattsson et al., 2019	327	AD	74.9 ± 7.8	45		46	675 ± 318	370 ± 146	36.9 ± 16
Melah et al., 2016	192	AD	61 ± 7.6	71.35	637 ± 316.4		738	307 ± 127	42 ± 15
Merluzzi et al., 2018	61	ADD	79 ± 5.3	38	1, 412 ± 799			725 ± 315	
Niikado et al., 2019	14	AD	71 ± 8.2	50	1, 335 ± 433		414 ± 124	380 ± 202	46.9 ± 16
Olsson et al., 2018	397	AD	71.2 ± 9.1	59.45	950 ± 426		134 ± 54	104 ± 54.55	36 ± 21
Paterson et al., 2018	156	AD	62.5 ± 3.2	58	1, 191 ± 557		311 ± 160	675 ± 171	86.4 ± 40
Pereira et al., 2017	65	AD	73.7 ± 7.6	47.69	1, 545 ± 586	43 ± 24		128 ± 56.3	42.9 ± 19
Pijnenburg et al., 2007	20	EAD	59 ± 4.4	50	950 ± 900		311 ± 104	523 ± 730	73 ± 72
Sánchez-Valle et al., 2018	22	AD-SMC	49.2 ± 10	59.09	1, 609 ± 469	25 ± 12	526 ± 267	871 ± 669	120 ± 93
Sjögren et al., 2001	22	AD	64.4 ± 7.7	31.82	569 ± 308		415 ± 147	725 ± 389	
Skillbäck et al., 2013	5,542	AD			3, 007 ± 6, 935		602 ± 286	504 ± 953	60 ± 33
Steinacker et al., 2018	26	AD	67 ± 8	57.69	1, 595 ± 1, 005	32 ± 16	545 ± 226	659 ± 268	78 ± 34
Vogt et al., 2018	40	AD	71.9 ± 8.6	40	1, 628 ± 935			722 ± 300	74.7 ± 27
Landqvist Waldö et al., 2013	20	AD	72 ± 10	65	415 ± 309		285 ± 92	630 ± 621.9	87 ± 58
Weston et al., 2017	48	AD-fam	40.5 ± 8.2	45.83		25 ± 12			
Weston et al., 2019	61	AD-fam	41.1 ± 8.1	50.82		15 ± 9			
Zerr et al., 2018	88	AD	71 ± 11	56.82	5, 538 ± 8, 887			557 ± 522	
Zetterberg et al., 2016	95	ADD	76 ± 3.2	44.21	1, 479 ± 632				
Zhou et al., 2017	187	AD	75.5 ± 7.4	48		51 ± 27			

*AD, Alzheimer's disease; ADD, AD dementia; AD-DS, AD with down syndrome; AD-fam, familial AD; AD-MCI, AD-mild cognitive impairment, AD-SMC, AD-symptomatic mutation carrier; CSF, cerebrospinal fluid; EAD, early onset AD; LAD, late onset AD; NFL, neurofilament light*.

### Meta-Analyses of Correlation Coefficients Between NFL and (1) AD Biomarkers and (2) MMSE Score

**Figure 2:**
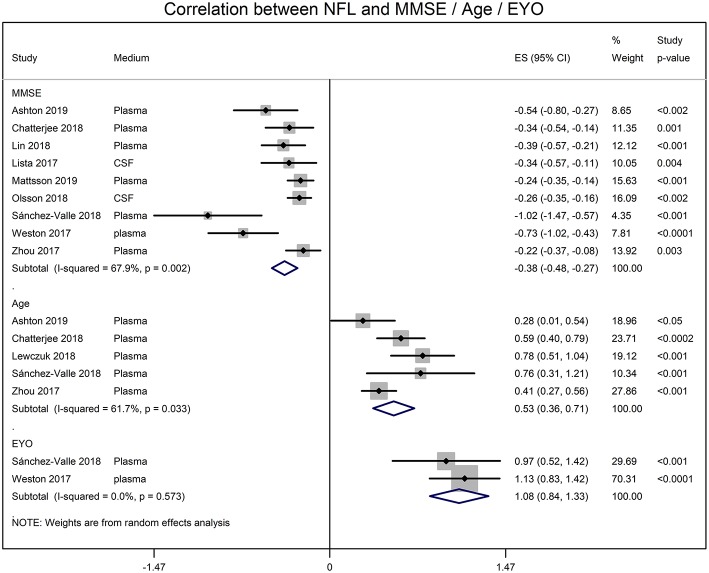
Forest graphs showing the meta-analyses of correlation coefficients (converted to z-scores) between NFL levels and MMSE (min-mental state examination) score, age, and EYO (estimated year of onset).

Meta-analyses of correlation coefficients reported by the included studies showed that serum NFL and CSF NFL levels correlated well (*r* = 0.59 [95% CI: 0.45, 0.71]; *p* < 0.0001). NFL levels significantly correlated with MMSE score (*r* = −0.345 [95% CI: −0.430, −0.254]; *p* = 0.0001), age (*r* = 0.485 [95% CI: 0.345, 0.611]; *p* = 0.00001) and estimated year of onset (*r* = 0.793 [95% CI: 0.686, 0.869]; *p* = 0.0001) (Figure 2).

**Figure 3:**
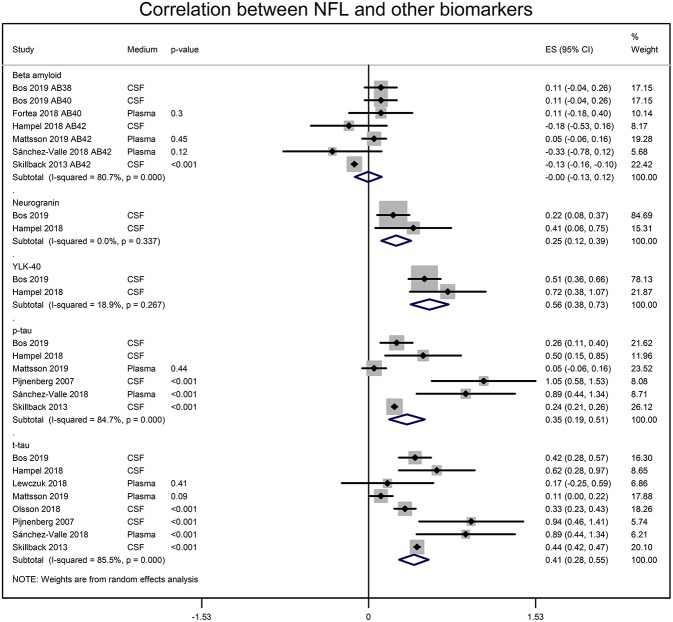
Forest graphs showing the meta-analyses of correlation coefficients (converted to z-scores) between NFL levels and biomarkers of AD. p-tau, phosphorylated tau, t-tau, total tau; YLK-40, and Chitinase-3-like protein 1.

NFL and the CSF biomarker of AD were also significantly correlated. CSF NFL concentrations significantly correlated with t-tau (*r* = 0.388 [95% CI: 0.273, 0.500]; *p* = 0.0001), p-tau (*r* = 0.336 [95% CI: 0.188, 0.470]; *p* = 0.00001), neurogranin (*r* = 0.245 [95% CI: 0.119, 0.371]; *p* = 0.001) and Chitinase-3-like protein 1 (YKL-40; r = 0.508 [95% CI: 0.363, 0.623]; *p* = 0.00001) (Figure 3). The CSF NFL concentration did not correlate with Aβ (0.00 [95% CI: −0.129, 0.119]; *p* = 0.937).

### Correlative Associations Measured From the Baseline Data of Included Studies

A correlation matrix drawn from the baseline value of biomarkers, age, and MMSE scores reported by the individual studies is presented in Table 2. In general, the blood and CSF concentrations of NFL were highly correlated. Age was positively associated with NFL levels but was negatively associated with CSF AD biomarkers. NFL concentrations were inversely associated with CSF biomarkers as well as MMSE scores.

**Table 2:** CSF Biomarker Correlation coefficient matrix.

	**CSF NFL**	**Serum NFL**	**Aβ-42**	**T-tau**	**P-tau**	**MMSE**
Age	0.132; *p* = 0.095	**0.706;** ***p*** **=** **0.0007**	−0.321; *p* = 0.110	−0.113; *p* = 0.089	–**0.217;** ***p*** **=** **0.002**	–**0.555;** ***p*** **=** **0.0005**
% Females	−0.040; *p* = 0.530	–**0.211;** ***p*** **=** **0.036**	**0.299;** ***p*** **=** **0.015**	–**0.194;** ***p*** **=** **0.015**	−0.073; *p* = 0.401	0.021; *p* = 0.604
CSF NFL		0.713; *p* = 0.0001	–**0.300;** ***p*** = **0.0001**	−0.073; *p* = 0.411	−0.058; *p* = 0.583	–**0.284;** ***p*** **=** **0.001**
Serum NFL			−0.143; *p* = 0.073	–**0.23;** ***p*** **=** **0.023**	−0.134; *p* = 0.094	–**0.623;** ***p*** **=** **0.008**
AB42				−0.124; *p* = 0.11	–**0.194;** ***p*** **=** **0.034**	**0.783;** ***p*** **<** **0.00001**
T-tau					**0.898;** ***p*** **<** **0.00001**	−0.108; *p* = 0.231
P-tau						−0.136; *p* = 0.094

*The bold values are statistically significant, p < 0.05*.

**Figure 4:**
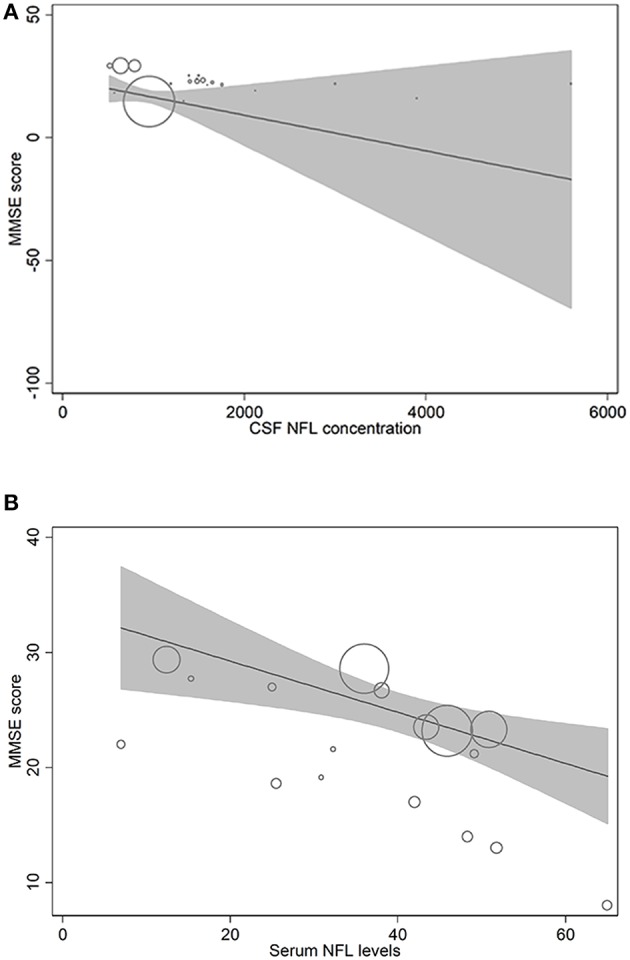
Meta-regression scatterplots showing the relationships between MMSE score and **(A)** NFL levels in CSF and **(B)** NFL levels in blood.

**Figure 5:**
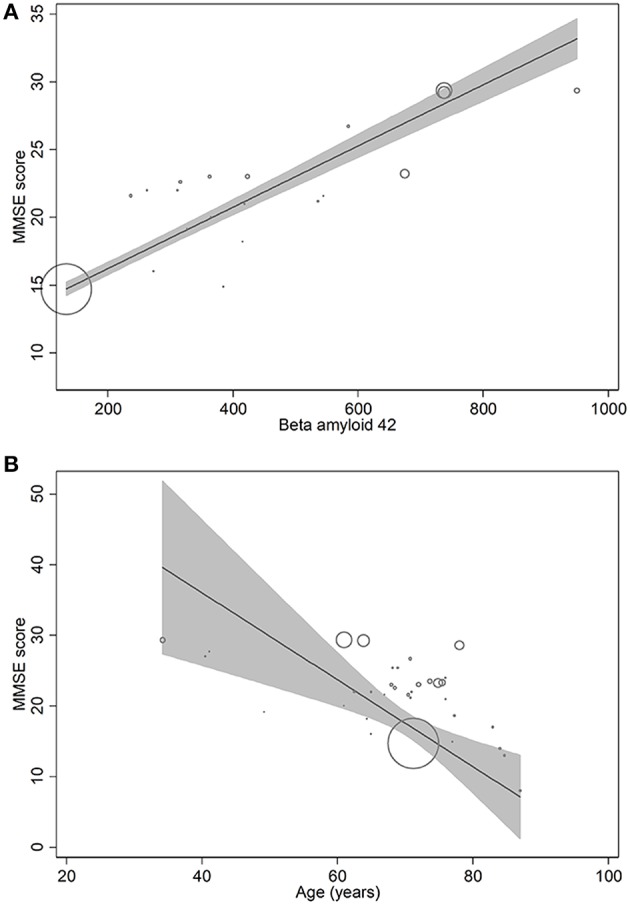
Meta-regression scatterplots showing the relationships between MMSE score and **(A)** beta-amyloid-42, and **(B)** age.

In metaregression analyses with MMSE scores as the dependent variable, MMSE scores were significantly inversely associated with blood NFL levels (metaregression coefficient (MC): −0.236 [95% CI: −0.400, −0.072]; *p* = 0.008) but had a statistically non-significantly inverse association with CSF NFL concentration (MC −0.001 [95% CI: −0.003, 0.001]; *p* = 0.220) (Figures 4A,B). MMSE scores were also significantly positively associated with Aβ-42 concentration in the CSF (MC 0.017 [95% CI: 0.010, 0.023]; *p* = 0.00001) but had asignificantly inverse association with age (MC −0.235 [95% CI: −0.360, −0.111]; *p* = 0.001) (Figures 5A,B). MMSE scores were statistically non-significantly associated with CSF t-tau levels (−0.002 [−0.009, 0.006]; *p* = 0.646) and CSF p-tau levels (−0.020 [95% CI: −0.091 0.051]; *p* = 0.567).

### Changes in NFL Levels in Individuals With AD Over Time

**Figure 6:**
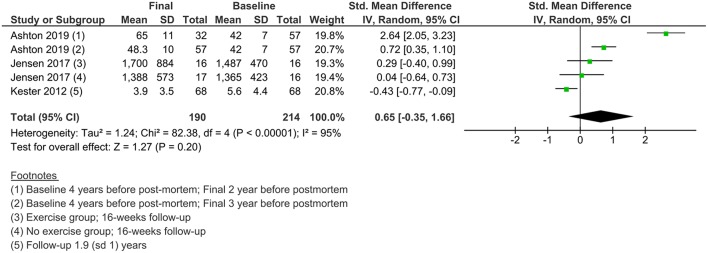
A forest graph showing the changes in NFL levels through time in AD patients.

Less data were available for the study of the changes in NFL levels over time. Three studies measured NFL levels in longitudinal designs with a follow-up duration of 16 weeks to 2 years. A meta-analysis of standardized mean differences showed no significant change from baseline to final values in NFL levels in AD patients (Figure 6).

## Discussion

In the present study, the meta-analyses of correlation coefficients reported by the individual eligible studies have shown that blood NFL and CSF NFL concentrations correlate well and that NFL levels correlate inversely with MMSE score but positively with age and estimated year of onset. While NFL levels and CSF Aβ concentrations were not correlated, other CSF biomarkers including t-tau, p-tau, neurogranin, and YLK-40 positively correlated with NFL. Many of these findings were also endorsed by the correlation and metaregression analyses of the present study for which baseline data of the included studies were used.

Recently, Mattsson et al. (2019) in a unified population consisting of normal individuals, those with mild cognitive impairment and those with AD also showed that plasma and CSF NFL concentrations significantly correlate. In this study, NFL levels were higher in individuals with ADD, which was also associated with cognitive deficits and the diagnostic accuracy of NFL for ADD was similar to established AD CSF biomarkers. Petzold et al. (2007) in a meta-analysis also found that the CSF levels of NFL were higher in individuals with AD than in normal individuals. A meta-analysis of 9513 participants from 47 datasets found that CSF NFL levels increased with age not only in healthy individuals but also in many patients with neurodegenerative diseases including AD. The authors suggested that age-specific reference values for CSF NFL should be used for the differential diagnosis (Bridel et al., 2018).

In the present study, we found that NFL levels correlated positively with age but CSF concentrations of Aβ, p-tau and MMSE scores correlated negatively with age. Yilmaz et al. (2017), reported that CSF NFL concentrations in 359 neurologically and psychiatrically healthy HIV negative individuals gradually increased from age 20 years (387 pg/ml) through 30 (525), 40 (713), 50 (967), 60 (1,313), 70 (1,781) to 80 years (2,417 pg/ml). In this study, the correlation coefficient between the log CSF NFL concentration and age was 0.77 (*p* < 0.0001).

In the present study, whereas CSF NFL levels were significantly correlated with t-tau, and p-tau, we could not find correlation between Aβ levels in CSF and CSF NFL levels. The presence of more amyloid plaques leads to lower Aβ levels in CSF, whereas higher neurofibrillary tangles causes higher tau CSF levels. Aβ, tau, p-tau or their ratios have associations with neurofibrillary tangles and neuritic plaques (Rosenmann, 2012).

We found a significant inverse correlation between serum NFL and MMSE scores not only in the meta-analyses of the correlation coefficient reported by the individual studies but also from cross sectional data pertaining to NFL levels and MMSE scores which we used to perform correlation matrix and metaregression. In addition to MMSE score, the NFL concentrations are found to correlate negatively with other assessment tools such as Clinical Dementia Rating Scale, the Recognition Memory Test (Weston et al., 2019) and the cognitive subscale of AD assessment scale (Mattsson et al., 2019).

Bos et al. (2019) who identified NFL as a strong predictor of cognitive decline in Aβ positive individuals suggested that NFL concentrations increase early in AD. Lewczuk et al. (2018) also found NFL levels to be higher during the early dementia stage than at the mild cognitive impairment stage in AD patients. Two studies included in the present meta-analysis reported a significant correlation between plasma NFL levels and the estimated year of onset (Weston et al., 2017; Sánchez-Valle et al., 2018). Because Aβ alone may not exclusively cause symptomatic AD, identification of downstream neuronal loss markers is highly desirable. One such candidate that could be used as a screening biomarker in pre-symptomatic trials is NFL (Weston et al., 2019).

A high diagnostic performance of plasma NFL to distinguish asymptomatic Down syndrome from Down syndrome with AD-dementia (AUC 0.95 [95% CI: 0.92, 0.98; sensitivity 90%; specificity 92%) was found by Fortea et al. (2018). In their study, plasma NFL levels were higher in individuals with Down syndrome in comparison with healthy controls suggesting that plasma NFL can help in the clinical diagnosis of cognitive impairment in complex cases.

More studies are required to elucidate the role of NFL in the differential diagnosis of related conditions. So far, de Jong et al. (2007) found that the CSF levels of NFL could be used to differentiate FTD from early AD but were unable to differentiate between late AD and DLB. Elevated CSF NFL levels were found by Skillback et al. (2014) in FTD and vascular dementia, and the lowest levels were in early AD individuals. In the present study, 10 of the included studies also reported NFL levels in individuals with FTD. Overall, in these studies the NFL levels were 1648 picogram/milliliter [931, 2366] in FTD and 1166 picogram/milliliter [689, 1642] in AD patients (weighted average [95% CI]). Moreover, the correlation coefficient between FTD and AD NFL levels in these studies was 0.966 which further strengthens the notion that NFL levels may be helpful in differentiating FTD from AD.

A crucial preventive strategy for AD would be to have timely neuroprotection before symptoms worsen and use of preventive medicine would be an important decision-making step for AD management. Although, NFL is unlikely to serve as a sole biomarker for the differential diagnosis of AD out from several neurodegenerative diseases with resembling NFL picture, its value in determining disease severity, predicting disease progression and forecasting prognosis should not be ignored. Moreover, formulating a treatment regimen and determining treatment response may also require NFL-based information. For example, differentiation between phosphorylated and non-phosphorylated forms of NFL (Hoglund et al., 2015) can be informative for optimal therapy.

Correlation coefficients reported by the individual studies show that that serum NFL levels correlate with CSF NFL concentrations, and NFL levels and MMSE scores are strongly negatively correlated, but NFL levels positively correlate with age. While the NFL levels and CSF Aβ concentrations were not correlated, other CSF biomarkers including t-tau, p-tau, neurogranin, and YLK-40 were positively correlated with CSF NFL concentrations. These findings were also endorsed by the correlations estimated from the mean values of the baseline data of included studies and metaregression analyses performed by using MMSE scores as the dependent variable. These correlations suggest that NFL may attain a role in AD diagnosis and prognosis, although future studies will be required to evaluate the NFL's precise role in the light of age-adjusted changes, AD risk analysis, and disease course.

## Data Availability

All datasets generated for this study are included in the manuscript/Supplementary Files.

## Author Contributions

MJ, LC, and YD wrote the manuscript and collected data. YD approved the manuscript.

### Conflict of Interest Statement

The authors declare that the research was conducted in the absence of any commercial or financial relationships that could be construed as a potential conflict of interest.
